# Towards a Design Toolkit of Informed Consent Models Across Fields: A Systematic Review

**DOI:** 10.1007/s11948-022-00398-x

**Published:** 2022-08-30

**Authors:** Iris Loosman, Philip J. Nickel

**Affiliations:** Department of Philosophy and Ethics, School of Innovation Sciences, Eindhoven University of Technology, PO Box 513, 5600 MB Eindhoven, The Netherlands

**Keywords:** Informed consent model, Systematic review, Biomedical ethics, Biobanking, Data ethics

## Abstract

In the 60+ years that the modern concept of informed consent has been around, researchers in various fields of practice, especially medical ethics, have developed new models to overcome theoretical and practical problems. While (systematic) literature reviews of such models exist *within* given fields (e.g., genetic screening), this article breaks ground by analyzing academic literature on consent models *across* fields. Three electronic research databases (Scopus, Google Scholar, and Web of Science) were searched for publications mentioning informed consent models. The titles, abstracts, and if applicable, full publications were screened and coded. The resulting data on fields, models, and themes were then analyzed. We scanned 300 sources from three databases to find 207 uniquely named consent models, and created a network visualization displaying which models occur primarily in one field, and which models overlap between fields. This analysis identifies trends in the consent debate in different fields, as well as common goals of consent models. The most frequently occurring consent models are identified and defined. The analysis contributes toward a cross-disciplinary “consent design toolkit” and highlights that there are more interrelationships between models and fields than are acknowledged in the literature. Where some models are designed to solve distinctively field-specific issues and are specific to biomedical ethics, some may be adaptable and applicable for other fields including engineering and design.

**Supplementary Information:**

The online version contains supplementary material available at 10.1007/s11948-022-00398-x.

## Introduction

*A team of software engineers is in the final stage of creating a new financial management app.* 1 *Their final challenge is to design and develop an informed consent process that will not only satisfy strict legal requirements, but also provide future app users with an opportunity to give their consent in a meaningful way. The app will contain several features that require users’ consent. It will connect to the users’ bank accounts, create overviews and analyses of users’ spending behavior on an ongoing basis, and provide personalized advice and coaching. Having collected this data for some time, it will help manage expenditures, check whether users might be eligible for benefits, and can even block part of the users’ funds for fixed expenses. Consent, in the app, is thus required for multiple and varying functionalities over a longer period of time. With the app, the team of engineers targets users looking for help in tackling financial issues. It can be downloaded by anyone who owns a smartphone. The team wonders whether providing a one-time click-to-agree box with some attached Terms of Agreement and a Privacy Statement will be sufficient. What other consent model would fit with the specific features of their case? Perhaps consent that can be altered at any time, or one that is divided into stages?*

The modern concept of informed consent has been around for over sixty years. During that time, it has been so extensively discussed that researchers, especially bioethicists, may sometimes feel they know everything about it. However, in the last few years, the topic has surged once again. Many new or alternative models have been developed in various fields from information technology to clinical studies, in order to overcome numerous theoretical and practical problems, some field-specific, some general. While informed consent continues to be considered desirable by most authors, many find flaws in what they term the “traditional model.”

What is referred to as the traditional model varies per setting. In research for example, informed consent was traditionally required for each study in which a human subject participates, also called “study-specific consent” (Mikkelsen et al., 2019). In care settings, consent traditionally takes place between a physician and a patient, whereby the latter is required to agree to a specific treatment proposed by the former. The traditional consent model is generally described in terms of autonomous authorization (e.g. Alblas et al., 2019; Grady, 2015; Schermer et al., 2014), defined as an autonomous action by a subject or patient to authorize a professional to either involve a subject in research or initiate a treatment for a patient, or both (Faden & Beauchamp, 1986). Informed consent is given if a subject or patient intentionally authorizes a professional to do X, with substantial understanding, and in substantial absence of control by others (Faden & Beauchamp, 1986).

Authors criticizing the traditional model call for a change in its practical realization, or even a reconceptualization of its fundamental ideals. Long-standing concerns, like low health literacy, or the unattainable ideal of being “fully informed” (Grady et al., 2017; Ploug & Holm, 2013), continue to be topics of discussion. Other more recent concerns arise with the application of informed consent to novel contexts and cases: for example, the unpredictability of consequences of data sharing and the difficulty of re-consent in data-intensive contexts, or the uninformed ticking of “agree boxes” in online environments (Custers, 2016). The traditional model of informed consent continues to be challenged from the perspective of many different fields far from its origin in bioethics, such as data science, law, and sexual ethics.

Over the past years, authors within these fields have been discussing *alternative consent models*, new and improved models to solve some aspect of their identified consent problems (Anderson et al., 2017; Burns et al., 2011; Cheung, 2018; see e.g. Kass et al., 2016). The number of alternative consent models is growing rapidly. Should we think of this proliferation as a “marketplace of ideas”, in which useful consent models get picked up, while others do not? Or, should we worry about this proliferation, as it complicates the debate and hinders good ideas from spreading?

As the scope of this debate continues to grow, it becomes difficult to get a clear overview of what is going on. Not every consent model tackles the same problem, nor does it always relate to existing models in different fields. Many models do not recur after their introduction, and many do not leave their field. Systematic reviews of consent literature have generally been performed *within* given domains or fields of practice (e.g., genetic screening) (Armstrong et al., 2017; da Silva et al., 2012; Gobat et al., 2015; Husedzinovic et al., 2015; van der Loos et al., 2015). There appears, therefore, to be a need for an overview of current literature on informed consent models *across* fields to recognize patterns at a higher order.

The goal of this review is to analyze literature on consent models *across* fields. This will increase knowledge of already available models and solutions in a first step towards a cross-disciplinary “consent design toolkit” useful to (ethicists of) engineering and design. Such a toolkit could inspire and inform the local application of consent models in cases like the one described at the beginning of this introduction. As a step toward the creation of such a toolkit, this review performs a broad sweep of existing literature on informed consent models and explores some of the main “tools” already out there. It provides an overview of the most commonly discussed consent models, brief definitional statements, and an informative representation of the current state of affairs within the consent discussion across fields. This tells us whether some concepts are already being shared across fields, and whether something could be gained through cross-disciplinary comparison and cross-fertilization. We hypothesize that similar consent problems occur across fields and solutions (or “tools”) proposed within these debates could prove beneficial to other fields as well. Creating a viable consent toolkit would help reduce unnecessary proliferation of models, and ensure that extensive work creating and validating consent models within specific contexts does not go to waste.

## Methods

### Definitions

For this study, we define consent models as any standardized mode or procedure by which an individual or a group agrees to a given course of action or treatment.

### Literature Search

The search protocol for the systematic literature search process and analysis was adapted from the Preferred Reporting Items for Systematic Reviews and Meta-Analyses (PRISMA), although due to the nature of this review not all items on the checklist were applicable (Moher et al., 2009). The review was conducted in five phases. In the first phase, articles written in English were found via three databases (Scopus, Web of Science, and Google Scholar), using a combination of the search terms “informed,” “consent” and “model,” searched in all fields.^2^^,^^3^^,^^4^^,^^5^ Date restrictions were not enforced. The databases were selected to ensure a wide range of search results from different fields, from (e.g.,) medicine, computer science, engineering, and law. For each search engine, only the first 100 search results sorted by relevance were included. Test searches with synonyms for “model” (e.g. “approach” and “procedure”) did not provide enough additional relevant sources to merit inclusion in the study.

In the second phase, all titles and abstracts were scanned to determine whether they were relevant to the study, i.e., containing a consent model that met the broad definition (see: Definitions).^6^ Relevant publications were scanned in full and their citations and the mentioned consent models logged. The remaining publications were assessed for mentioning a “traditional consent model” and for mentioning other potential fields of application. Additional informal notes were kept on themes in the texts and (where applicable) reasons for abandoning the traditional model. To avoid bias, substantial parts of phases one and two were independently repeated by the second author, followed by comparative discussion.

The third phase of the review identified consistent field labels for all items using a bottom-up iterative analysis. The authors discussed and agreed upon consistent names for the identified fields such as biobanking, newborn screening, and critical care research. Repeating the same iterative process, these fields were then further categorized, to form ten meta-level field labels. The ten meta-field labels were used to sort the articles according to field, and to count how many models were found within them (see Additional file 1).

In phase four, a network visualization (Fig. 4) was created for those consent models mentioned at least twice. In the fifth and final stage, an overview of the definitions and primary fields of the fourteen most frequently mentioned consent models was formulated. The resulting overview can be found in Table 2. Definitions have been formulated based on the publications included in the final list.

This literature study did not require IRB approval, as it did not involve contact with persons.

## Results

### Identified Consent Models

**Fig. 1:**
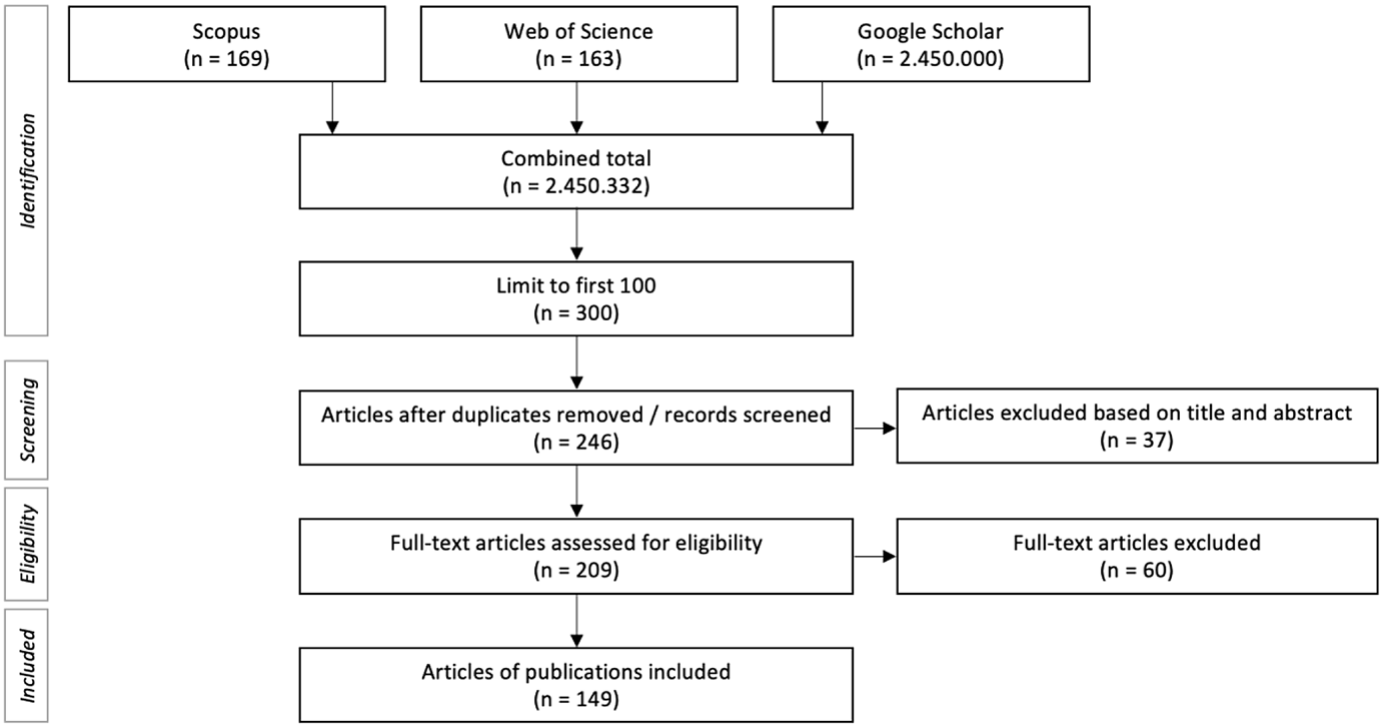
Flow chart of selection and inclusion of publications

A total of N = 300 publications was identified for review across the three databases. After applying the exclusion criteria, the final sample of publications was N = 149 (Fig. 1). The final number of consent models within these 149 papers was 207, some of which were non-unique (i.e. synonyms or combinations of other models). Together, the 207 models were mentioned 461 times in the 149 publications.

**Fig. 2:**
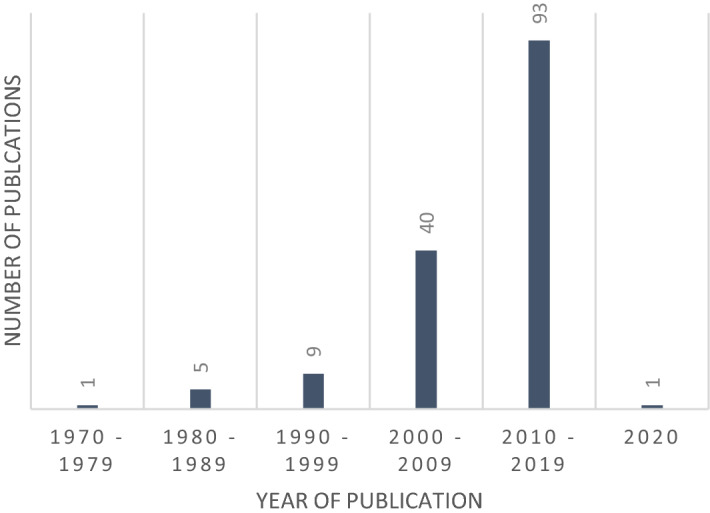
Number of articles in sample (n = 149) sorted by year of publication

The results in the final publication sample of 149 were published between 1977 and 2020. Figure 2 depicts the distribution of the publications over the years in between. The figure indicates that by far the most results in the sample were published between 2010 and 2019 (93 publications).

The iterative process of assigning field labels to the 149 publications resulted in an overview of the quantity of publications per meta-field. Table 1 shows the number of publications per field.

**Table 1:** Number of included publications per field

Field	No. of publications
Biobanking research/genetics and genomics research	47
Clinical care other/including telehealth and nursing	28
Other clinical research	20
Health informatics	17
Critical care/critical care research	11
Screening/including family and prospective parents	11
ICT and data	6
Non-interventional medical research	4
Organ donation/transplantation	4
Other	1

**Fig. 3:**
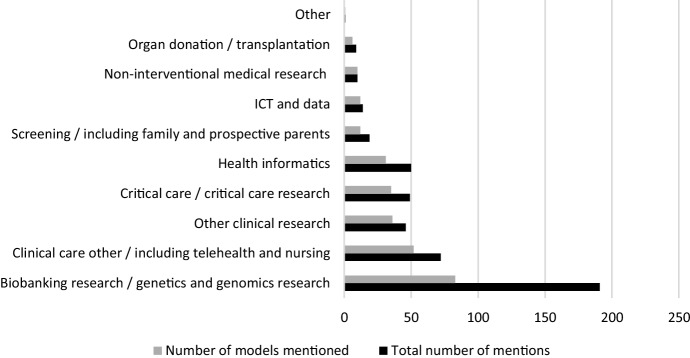
Number of informed consent models mentioned per field and total number of mentions

To determine how intensively consent models were discussed within the fields, the list of the 207 consent models (including the “orphan models”), was combined with the list of fields in which they were mentioned, and the amount of times they were mentioned. Figure 3 shows that by far the most of the 207 model names were mentioned in the field of *Biobanking research/genetics & genomics research*, with 83 models mentioned. These 83 models were mentioned 191 times in the publications in this field, as indicated by the lower bar.

**Fig. 4:**
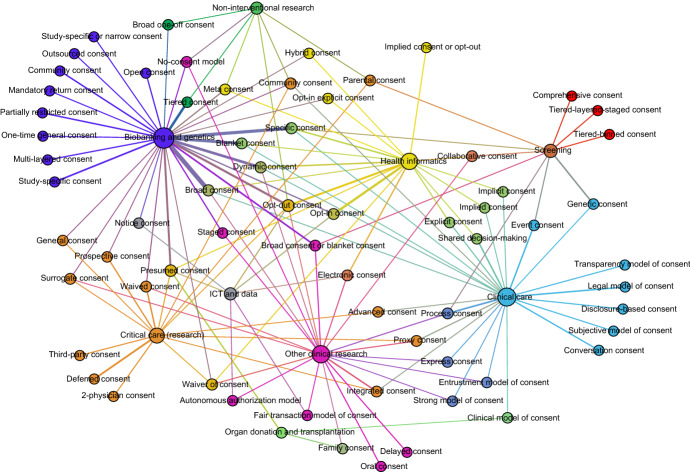
Network analysis of consent models

Applying an additional exclusion criterion resulted in the exclusion of 142 “orphan models” from further analysis. These models did not recur in other publications in our sample. The relationships between the remaining 65 models (mentioned in two or more publications) were explored by creating a network analysis (Fig. 4).

In the network analysis the 10 fields were combined with the 65 models, to discover connections between models and fields. The network analysis shows which models occur primarily within one field, and which models overlap between fields. The figure shows the “stand-alone” models towards the edges (e.g. study-specific consent and outsourced consent in the top-right corner), and the more interconnected models towards the center of the network (e.g. opt-in consent in the middle). Thicker lines between fields and models mean there are more publications within this field that mention this model.

### Other Findings

Fourteen consent models are mentioned at least six times in the final count (see Additional file 2). Drawing from the publications within the sample, short typical definitional statements of these models were formulated, along with remarks on the models’ field(s) of earliest occurrence within the results (Table 2).

**Table 2:** Frequently occurring consent models and typical definitions^a^

Consent model	Definition	Field(s) of early occurrences^b^
Broad	Individuals “prospectively agree to their samples and … information being used in any future research” (Simon et al., 2011). This amounts to “consent to a particular kind of governance arrangement” in which “somebody else, usually [a] governing body … decide[s] how to use [a person’s] sample or data” (Sheehan, 2011). Nb. some studies equate broad and blanket consent	Biobanking
Opt-out	Information is provided at a given moment, with a default of participation. Individuals are expected actively to express their intentions in order to be *excluded* (Coiera & Clarke, 2004; Simon et al., 2011)	IT and health informatics
Dynamic	Digital platforms or other “modern communication strategies” (Steinsbekk et al., 2013) are used to inform individuals about, and allow fine-grained preference-setting for participation in, new or additional research projects in which their sample or data might be (re)used (Stein & Terry, 2013)	Biobanking
(Study-)specific	Individuals are (re)contacted about each instance in which their sample or data is (re)used, provided with adequate information regarding the benefits and risks of participation on that occasion, and given the opportunity to be included or excluded (Allen & Mcnamara, 2011)	Biobanking
Blanket	Often equated with broad consent (e.g., Simon et al., 2011). When distinguished from broad consent, it is understood as granting an *unqualified* license for participation (Thompson & McNamee, 2017)	Biobanking
Presumed	Participation is the default. There is no moment at which consent is granted. However, information is provided in a general way, e.g., by way of a poster or leaflet, and individuals often have the right to opt out (Gefenas et al., 2012)	Early occurrences diverse
Opt-in	Contrasted with “Opt-out” (see above). Information is provided at a given moment, with a default of *non-*participation. Individuals are expected to express their intentions in order to be *included* (Simon et al., 2011)	IT and health informatics
Tiered	Individuals select from a preformulated menu of possible areas of participation and parties to involve when deciding whether and how to participate (Bunnik et al., 2014)	Biobanking and genetic screening
Process	In the context of an ongoing communicative relationship between individuals and professionals, there is mutual information exchange and assent to an intervention or other course of action (Lidz et al., 1988; Usher & Arthur, 1998)	Clinical care
Waived and Waiver of consent	Under special conditions recognized by a legal entity such as an IRB as grounds for an exception, no consent is required for participation (da Silva et al., 2012; Duffett et al., 2011)	Early occurrences diverse: critical care, secondary use research
Deferred	In situations where an individual cannot give consent and no alternative (e.g., a proxy) is available, the decision whether to continue to participate takes place after participation has already been initiated (Burns et al., 2011)	Critical care
Event	Contrasted with “Process” (see above). Legal authorization is granted at a single moment, on the basis of specified information that has been transmitted to the individual beforehand (Delany, 2008; Lidz et al., 1988)	Clinical care
Integrated	Information about participation is delivered and assent is given as a part of an ordinary transaction between individual and professional, e.g., during a clinical encounter (Kim & Miller, 2014)	Pragmatic trials
Proxy	Consent is provided on behalf of an individual who lacks capacity, sometimes referred to as surrogate consent (Armstrong et al., 2017; van der Loos et al., 2015)	Early occurrences diverse: clinical care, critical care

^a^The terms “participation” or “inclusion” in these definitions are used to refer to the general object of consent. In this way the definitions depend less on field-specific elements

^b^This refers to early occurrences in the sources included in the review; earlier historical or etymological occurrences outside the scope of the review could lie in a different field

From the final 149 publications in the sample, 60 publications explicitly describe their discussed consent model(s) in relation to, as an alternative to, or as opposed to, a “traditional consent model” (e.g. Semprini et al., 2017). The number of publications that only implicitly discussed a traditional consent model (e.g. describing “alternative models,” but not explicitly addressing their counterpart), or that used a different term (e.g. “bioethics model of consent” in McKneally & Martin, 2000) is 36. 53 publications neither mention nor imply a traditional consent model.

Of the 149 publications, fifteen comment on other potential applications for their discussed model(s). These potential applications are often closely related to the situation for which the model was originally introduced. For example, a model in a specific genetics research project that could be applied in fields with similar research participation issues (Kelly et al., 2015). Another example is a model in genetic screening that could be applied in other future forms of genetic testing or screening in both commercial and clinical settings (Bunnik et al., 2014).

### Limitations

Despite thorough test runs, the inclusion criteria and search strings selected for this review limited the inclusion of potentially relevant research. A number of papers raised relevant issues but did not meet all the inclusion criteria. Using other databases, or including more than the first 100 results, might have included additional studies and reflected the current literature more broadly. The data show that the discussion of consent models has surged over the last decade, but it could be that different wording was previously used to discuss the same phenomena.

Some consent model names that were included appeared to be non-unique, either because they could be considered synonyms for the same model (e.g. “one-time consent” and “one-off consent”), or because they were names consisting of several models the author(s) described in one combined name (e.g. “broad consent or blanket consent”). In our analysis these models were left *as is*, to safeguard objectivity. However, this did increase the number of models in our results.

As the search for consent models in the full texts was performed manually it is possible that a model was incidentally overlooked, even though great care was taken to avoid this.

## Discussion

### Connections Between Models

Although only fifteen of the 149 publications explicitly mention other potential applications for their discussed model(s), the network visualization shows there are more interrelationships between models and fields than are explicitly being acknowledged (Fig. 4).

Some models are uniquely introduced for one field and not discussed elsewhere. This is the case with *deferred consent* in Critical care/critical care research, for example (e.g. Petriş et al., 2015). For reasons of urgency inherent to emergency and critical care itself, participants in research are temporarily unable to provide full consent to participation. This model defers the full consent process until the patient has recovered sufficiently (Table 2).

Other models are discussed in several fields. *Presumed consent*, for example, occurs in publications in five different fields (see Additional file 2). The connectedness of this model suggests it tackles a problem that occurs in several fields. In other cases, models are specifically designed to solve a consent problem in one field but are explicitly linked with models in other fields. An example is the *tiered-layered-staged consent model,* a model first introduced in Bunnik et al. (2013), found in the field of *Screening* (Alblas et al., 2019, see Additional file 2). Even though this model did not appear in other fields, “tiered”, “layered”, and “staged” consent occur elsewhere (see Additional file 2). The combination of these elements into a unique and field-specific consent model appears to make use of cross-pollination, while tailored to a specific application.

Orphan models in the dataset are of various types. A few examples help to illustrate. The *broad (unspecified) consent model* (Allen & Mcnamara, 2011) is just a minor variation on broad consent, the most frequently occurring model. By contrast, the *emergency trial consent model* (Iwanowski et al., 2008) is invented by the authors to cover a logical disjunction of models permitted by “good clinical practice” guidelines. The *wiki-governance model* (referencing Dove et al., 2012; Kelly et al., 2015) has been discussed in the biobanking literature, but it is usually described as a governance model rather than a consent model (cf. Steinsbekk et al., 2013). The *semantic model of consent* (Fatema et al., 2017) is an ontology meant to facilitate the management of consent permissions for digital datasets.

The variety and proliferation of consent models suggest that a repository of innovative models and variations on existing models can serve as a “marketplace of ideas” that can help to address new consent challenges. Yet a disadvantage is that research and communication effort is wasted on numerous models and variations that are not retained or found useful by practitioners. Terminological confusion and a proliferation of models can lead to empirical studies of consent that talk past one another and cannot easily be aggregated in meta-analyses or compared across institutional contexts.

### Connections Between Fields

Some of the fields appear to have a high degree of interconnectedness with other fields, as is evidenced by their central location in the network analysis (Fig. 4). The fields *ICT and data*, *Health informatics* and *Other clinical research* showed up more towards the center of the network. A possible explanation for this interconnectedness is that these fields combine aspects of other related fields. An example of such a combination of aspects can be found in the field *Health informatics*. The use of electronic patient records in health care combines aspects of ICT, health data, and clinical care (e.g. Spencer et al., 2016). It seems logical to draw on closely related fields for solutions to new and emerging consent problems. The introduction of new technologies into existing fields can have a similar effect. With the introduction of telehealth into health care, for example, it is not immediately clear if informed consent still applies similarly (Chouinard & Scott, 2009). Though the goal of providing care to patients remains equal, the means to deliver it now involves digital communication of sensitive data. Models in other fields, designed to tackle issues with transmission of health data, may prove informative here (Chouinard & Scott, 2009).

Figure 3 shows that most models were mentioned within the field *Biobanking research/genetics & genomics research*. The fact that a large number of the consent models is found in biobanking is unsurprising, as an increasing number of bioethics publications about biobanking have appeared over the last 15 years, with consent as one of the major topics of discussion (Coppola et al., 2019). The biobanking consent debate revolves around how to best handle enrollment of participants, where subsequent use of samples and data in other studies has high utility but is of an unpredictable nature (Mikkelsen et al., 2019). An often-mentioned reason for deviating from the more traditional study-specific research consent model is that the practicalities of research involving biobanks (e.g. the size of the cohorts and the frequency of new studies) increasingly hinder this kind of consent (Mikkelsen et al., 2019).

A number of features of biobanking, for example data-intensiveness, multiplicity of research purposes, potential for re-use of data and samples, etc. are not unique to this field. This may be one explanation for why biobanking has connections with consent models in related fields with similar features (e.g. *Health informatics*, *ICT and data*, and *Other clinical research*; see Fig. 4). This study revealed some models that were highly connected between fields, for example *opt-in consent* and *dynamic consent*. However, although there were several highly connected models in the meta-field of *Biobanking research/genetics & genomics research*, its location in the network analysis is towards the edge, which suggests a more independent scholarly discussion compared to other more centrally-located fields.

### Consolidating Existing Knowledge of Models

The 83 models mentioned in *Biobanking research/genetics & genomics research* were mentioned a relatively high number of times (191), as compared to the ratio of models and mentions in the other fields. The high total number of mentions of the models suggests that the debate is most intensive in this field, with many publications discussing and comparing more than one model. The data in our pool confirm that a high number of publications within this field discuss, compare or contrast a relatively high number of established consent models before zooming in on a particular preferred consent model (see Additional file 3). This suggests that within this field, relative to other fields, existing knowledge is consolidated more, as well as assessed at a meta-level.^7^

Although this means that in the field of *Biobanking research/genetics & genomics research* newly introduced consent models are often better embedded in the existing landscape of models in this field, we still observe a high number of stand-alone models (Fig. 4), as well as orphan models not recurring in other publications (see Additional file 2).

Zooming out to the general dataset, a third of the 149 publications (n = 58) explicitly mentioned a traditional consent model before introducing one or multiple alternative consent models. The fact that a third of all publications in the dataset mentioned a traditional consent model in relation to the alternative(s) is a positive sign of integration of the latter in the existing discussion, but it does not necessarily mean that it also connect(s) to *other* alternatives. The network analysis revealed 142 models that did not recur in more than one publication in the dataset. This suggests that there is a proliferation of models that are never picked up on in the debate, either within or between fields.

Thus, although it is a positive development within the field of *Biobanking research/genetics & genomics research* that existing knowledge is consolidated more often, when we look *across* fields, not all models are picked up on and discussed again. This is unfortunate, as consolidation of existing knowledge of models could decrease the chance that new models are subject to consent problems other authors have already pointed out elsewhere. It would ensure authors within a specific field are in conversation with each other, which helps to further the debate. This in turn could increase chances of retaining useful models. Looking beyond specific fields, there are models and solutions outside of isolated debates. Here it would be worth ‘crossing borders’ and placing these solutions in our proposed design toolkit. Such a toolkit would increase opportunities for models to be picked up by other fields where they might be applied or developed further to solve similar problems.

### Beyond Isolated Efforts

The consent literature would benefit from the realization that there is much outside the field of biobanking that deserves a place in our collective toolkit. As fields move away from traditional models, it is important to observe developments across fields that may prove informative for local consent solutions. There are many models on the market that might be beneficial beyond their field of origin. A recent example is the *meta consent* model, which lets people design how and when they would like to be presented with a consent request on the (secondary) use of their health data and biological material (Ploug & Holm, 2016). The model shows potential for application in other consent-intensive fields and for different media (e.g., a smartphone app: Ploug & Holm, 2017). A related example is the *dynamic consent* model, which utilizes a personalized communication interface through which research participants can manage and tailor their consent preferences (Kaye et al., 2015). Although it originated the field of biobanking, it is increasingly described as holding potential for other fields in which ongoing communication and engagement are similarly central, such as clinical care and digital health (Teare et al., 2021). These examples highlight the fact that as healthcare and advanced digital technologies become more intertwined, innovations from disparate fields need to share insights: for example, by using solutions from digital technologies to automate the documentation of consent, providing secure, authenticated access to the scope and status of consent by users, administrators and professionals; or in the opposite direction, by using solutions from data-intensive medical research for other ICT applications involving fitness and wellness more broadly construed.

Another example is that of *process consent*, a model that originated in clinical care, and describes consent in terms of exchange of information and assent in the ongoing communicative relationship between individual and professional (see Table 2). Consent as integral to an ongoing relationship is relevant far beyond the field of medicine. This idea might prove beneficial for fields in which there are similar ongoing and developing relationships between individual and professional, for example between mobile health application user and data processor, or in the application of persuasive technology.

A cross-disciplinary consent design toolkit, for example in the form of a repository or search tool, could ensure the accessibility of such efforts. Existing efforts to provide comprehensive guides to informed consent within specific fields, such as the comprehensive work on an eConsent design for independently administered consent in mHealth studies (Doerr et al., 2017), or the Sage Bionetworks ‘Elements of Informed Consent’^8^ toolkit for researchers working with study participants, could be united in such a cross-disciplinary toolkit. That way, researchers across fields could build upon existing knowledge and solutions, adopting and further developing promising solutions, rather than re-inventing them.

## Conclusion

Our main observations of the data on consent models may be summarized as follows:

- There is not much cross-fertilization between fields. Some models are discussed independently in different fields, some are isolated to a single field, and there are many orphan models.
- At the same time, a network analysis reveals that some fields such as *ICT and data* do have strong links with many other related fields.
- The most frequently reused consent models are in the field of biobanking, indicating extensive consolidation of these models. However, there are widely used models from other fields which appear to solve different problems with “traditional” consent.

The challenges to obtaining valid informed consent, even when they are deeply connected to a specific field, often have a general aspect that can reappear. For example, in the field of critical care, an inherent problem is that the consenting subject in the emergency room is unconscious or in a crisis mode. Their decisions are critically important, but they (temporarily) lack decision making capacity. Similar challenges could in principle arise elsewhere, for example when somebody uses a smartphone application to report a violent crime of which they are the victim, requiring a consent decision about data-sharing at a crisis moment.

For these reasons, there appears to be great promise in assembling a toolkit for designing and adapting existing consent models in new contexts. The results of this study provide a resource for those considering what consent model might best solve emerging problems in (e.g.,) mobile health, the use of public health apps to control pandemics, and apps for financial planning such as the one with which we started the paper. Rather than proliferating the market of ad hoc consent models, and re-inventing the wheel, practitioners and researchers could find consent tools to address new design problems with features similar to existing problems. This review study reveals connections between different consent debates, and the models they contained, as a first step in this direction.

Future research may be directed at providing in-depth analyses of (recurring elements of) consent models across fields, analyses of the different consent challenges to which alternative consent models respond, mapping trends and themes, or mapping design requirements to solutions using the whole kit of established tools. Performing additional thematic analyses on reasons for deviating from traditional models may prove helpful in finding and solving shared consent problems between fields.

We envision an open source, dynamic design toolkit in which consent solutions can be added, or altered, along with references to the evidence base supporting them. The next step to create this toolkit could be to analyze and map consent models by the features of the context to which they are responding. These features can for example concern the (envisioned) user, the situation, or the technology that the consent model will be used with. Searching the toolkit for a specific feature could then provide a selection of (templates of) models that those looking for a consent solution can test out in their specific field of application, for example through a process of reflective design.

In this paper, we have shown that the landscape of consent models is complex when viewed at a broader level of resolution. Although researchers, practitioners, and designers know a great deal about consent, they can learn even more by peering over the garden walls separating seemingly discrete fields. A shared toolkit of consent models would help serve the underlying ethical and legal aims that the practice is meant to protect and promote.

## Supplementary Information

Below is the link to the electronic supplementary material.

Additional file 1. Field names. List of meta-fields and sub-fields.

Additional file 2. All models and number of times mentioned per field. Large table with all models found, sorted by number of mentions, per field.

Additional file 3. Publications and found models. Full list of publications included in the study sorted by field. List contains all models found per publication, and whether the publication contained mention of a ‘traditional model’ and other potential application fields (beyond field of publication).

## Footnotes

1 Example based on ‘Buddy Payment’; a debt prevention app that helps users pay and manage their bills. See https://buddypayment.nl.

2 After a test run the term “transgender” was explicitly excluded from the search terms, as the test revealed that “consent models” in relation to this topic had a different meaning and were connected to a different debate (see e.g. Deutsch, 2012). This decision had no discriminatory purpose whatsoever. Carefully comparing the results revealed that no relevant sources containing the term “transgender” were excluded.

3 Search string Scopus: (TITLE-ABS-KEY (informed AND consent) AND TITLE-ABS-KEY ("consent model" OR "model of consent") AND NOT TITLE-ABS-KEY (transgender)) AND (LIMIT-TO (LANGUAGE, "english")).

4 Search string Web of Science: Consent AND model AND ("consent model" OR "model of consent" OR "model of informed") NOT transgender) (English).

5 Search string Google Scholar: informed consent model OR consent model OR model of consent -transgender.

6 *Assent* is excluded from the models, but is mentioned often in the included texts (especially in critical care research). Although assent could conceivably be understood as a manner of agreeing to a proposal in accordance with our definition, it is more consistent with usage to consider it as an element within a consent model.

7 Examples of such articles are McGuire and Beskow (2010) and Jurate et al. (2014).

8 This toolkit, written by Sarah Moore and Megan Doerr, can be accessed through: https://sagebionetworks.org/tools_resources/elements-of-informed-consent/.

## References

[CR1] Alblas M, Schermer M, Vergouwe Y, Bolt I (2019). Autonomy challenges in epigenetic risk-stratified cancer screening: How can patient decision aids support informed consent?. Journal of Personalized Medicine.

[CR2] Allen J, Mcnamara B (2011). Reconsidering the value of consent in biobank research. Bioethics.

[CR3] Anderson EE, Newman SB, Matthews AK (2017). Improving informed consent: Stakeholder views. AJOB Empirical Bioethics.

[CR4] Armstrong S, Langlois A, Laparidou D, Dixon M, Appleton JP, Bath PM, Snooks H, Siriwardena AN (2017). Assessment of consent models as an ethical consideration in the conduct of prehospital ambulance randomised controlled clinical trials: A systematic review. BMC Medical Research Methodology.

[CR5] Bunnik EM, Janssens ACJW, Schermer MHN (2013). A tiered-layered-staged model for informed consent in personal genome testing. European Journal of Human Genetics.

[CR6] Bunnik EM, Janssens ACJW, Schermer MHN (2014). Informed consent in direct-to-consumer personal genome testing: The outline of a model between specific and generic consent. Bioethics.

[CR7] Burns KEA, Magyarody NM, Duffett M, Nisenbaum R, Cook DJ (2011). Attitudes of the general public toward alternative consent models. American Journal of Critical Care.

[CR8] Cheung ASY (2018). Moving beyond consent for citizen science in big data health research. Northwestern Journal of Technology and Intellectual Property.

[CR9] Chouinard I, Scott RE (2009). Informed consent for videoconsultations in Canada. Journal of Telemedicine and Telecare.

[CR10] Coiera E, Clarke R (2004). e-Consent: The design and implementation of consumer consent mechanisms in an electronic environment. Journal of the American Medical Informatics Association.

[CR11] Coppola L, Cianflone A, Grimaldi AM, Incoronato M, Bevilacqua P, Messina F, Baselice S, Soricelli A, Mirabelli P, Salvatore M (2019). Biobanking in health care: Evolution and future directions. Journal of Translational Medicine.

[CR12] Custers B (2016). Click here to consent forever: Expiry dates for informed consent. Big Data & Society.

[CR13] da Silva MEM, Coeli CM, Ventura M, Palacios M, Magnanini MMF, Camargo TMCR, Camargo KR (2012). Informed consent for record linkage: A systematic review. Journal of Medical Ethics.

[CR14] Delany C (2008). Making a difference: Incorporating theories of autonomy into models of informed consent. Journal of Medical Ethics.

[CR15] Deutsch MB (2012). Use of the informed consent model in the provision of cross-sex hormone therapy: A survey of the practices of selected clinics. International Journal of Transgenderism.

[CR16] Doerr M, Maguire Truong A, Bot BM, Wilbanks J, Suver C, Mangravite LM (2017). Formative evaluation of participant experience with mobile econsent in the app-mediated Parkinson mPower study: A mixed methods study. JMIR MHealth and UHealth.

[CR17] Dove ES, Joly Y, Knoppers BM (2012). Power to the people: A wiki-governance model for biobanks. Genome Biology.

[CR18] Duffett M, Burns KE, Kho ME, Lauzier F, Meade MO, Arnold DM, Adhikari NKJ, Lamontagne F, Cook DJ (2011). Consent in critical care trials: A survey of Canadian research ethics boards and critical care researchers. Journal of Critical Care.

[CR19] Faden R, Beauchamp T (1986). A history and theory of informed consent.

[CR20] Fatema, K., Hadziselimovic, E., Pandit, H., Debruyne, C., Lewis, D., & O’sullivan, D. (2017). Compliance through informed consent: Semantic based consent permission and data management model. In *CEUR workshop proceedings, 1951*.

[CR21] Gefenas E, Dranseika V, Serepkaite J, Cekanauskaite A, Caenazzo L, Gordijn B, Pegoraro R, Yuko E (2012). Turning residual human biological materials into research collections: Playing with consent. Journal of Medical Ethics.

[CR22] Gobat NH, Gal M, Francis NA, Hood K, Watkins A, Turner J, Moore R, Webb SAR, Butler CC, Nichol A (2015). Key stakeholder perceptions about consent to participate in acute illness research: A rapid, systematic review to inform epi/pandemic research preparedness. Trials.

[CR23] Grady C (2015). Enduring and emerging challenges of informed consent. New England Journal of Medicine.

[CR24] Grady C, Cummings SR, Rowbotham MC, McConnell MV, Ashley EA, Kang G (2017). Informed consent. New England Journal of Medicine.

[CR25] Husedzinovic A, Ose D, Schickhardt C, Fröhling S, Winkler EC (2015). Stakeholders’ perspectives on biobank-based genomic research: Systematic review of the literature. European Journal of Human Genetics: EJHG.

[CR26] Iwanowski P, Budaj A, Członkowska A, Wąsek W, Kozłowska-Boszko B, Olędzka U, Masełbas W (2008). Informed consent for clinical trials in acute coronary syndromes and stroke following the European Clinical Trials Directive: Investigators’ experiences and attitudes. Trials.

[CR27] Jurate S, Zivile V, Eugenijus G (2014). Mirroring‘ the ethics of biobanking: What analysis of consent documents can tell us?. Science and Engineering Ethics.

[CR28] Kass N, Faden R, Fabi RE, Morain S, Hallez K, Whicher D, Tunis S, Moloney R, Messner D, Pitcavage J (2016). Alternative consent models for comparative effectiveness studies: Views of patients from two institutions. AJOB Empirical Bioethics.

[CR29] Kaye J, Whitley EA, Lund D, Morrison M, Teare H, Melham K (2015). Dynamic consent: A patient interface for twenty-first century research networks. European Journal of Human Genetics: EJHG.

[CR30] Kelly SE, Spector TD, Cherkas LF, Prainsack B, Harris JM (2015). Evaluating the consent preferences of UK research volunteers for genetic and clinical studies. PLoS ONE.

[CR31] Kim SYH, Miller FG (2014). Informed consent for pragmatic trials—The integrated consent model. New England Journal of Medicine.

[CR32] Lidz CW, Appelbaum PS, Meisel A (1988). Two models of implementing informed consent. Archives of Internal Medicine.

[CR33] McGuire AL, Beskow LM (2010). Informed consent in genomics and genetic research. Annual Review of Genomics and Human Genetics.

[CR34] McKneally MF, Martin DK (2000). An entrustment model of consent for surgical treatment of life-threatening illness: Perspective of patients requiring esophagectomy. Journal of Thoracic and Cardiovascular Surgery.

[CR35] Mikkelsen RB, Gjerris M, Waldemar G, Sandøe P (2019). Broad consent for biobanks is best—Provided it is also deep. BMC Medical Ethics.

[CR36] Moher D, Liberati A, Tetzlaff J, Altman DG (2009). Preferred reporting items for systematic reviews and meta-analyses: The PRISMA statement. BMJ (Online).

[CR37] Petriş AO, Cimpoeşu DC, Ungureanu D (2015). What’s new in ethics of cardio-pulmonary resuscitation research: Too little time and too many rules?. Intensive Care Medicine.

[CR38] Ploug T, Holm S (2013). Informed consent and routinisation. Journal of Medical Ethics.

[CR39] Ploug T, Holm S (2016). Meta consent—A flexible solution to the problem of secondary use of health data. Bioethics.

[CR40] Ploug T, Holm S (2017). Eliciting meta consent for future secondary research use of health data using a smartphone application—A proof of concept study in the Danish population. BMC Medical Ethics.

[CR41] Schermer BW, Custers B, van der Hof S (2014). The crisis of consent: How stronger legal protection may lead to weaker consent in data protection. Ethics and Information Technology.

[CR42] Semprini A, Hills T, Braithwaite I, Weatherall M, Beasley R (2017). A priori consent within pragmatic randomised controlled trials: A web-based survey of statin use in primary care. BMJ Innovations.

[CR43] Sheehan M (2011). Can broad consent be informed consent?. Public Health Ethics.

[CR44] Simon CM, L’Heureux J, Murray JC, Winokur P, Weiner G, Newbury E, Shinkunas L, Zimmerman B (2011). Active choice but not too active: Public perspectives on biobank consent models. Genetics in Medicine.

[CR45] Spencer K, Sanders C, Whitley EA, Lund D, Kaye J, Dixon WG (2016). Patient perspectives on sharing anonymized personal health data using a digital system for dynamic consent and research feedback: A qualitative study. Journal of Medical Internet Research.

[CR46] Stein DT, Terry SF (2013). Reforming biobank consent policy: A necessary move away from broad consent toward dynamic consent. Genetic Testing and Molecular Biomarkers.

[CR47] Steinsbekk KS, Kare Myskja B, Solberg B (2013). Broad consent versus dynamic consent in biobank research: Is passive participation an ethical problem. European Journal of Human Genetics.

[CR48] Teare H, Prictor M, Kaye J (2021). Reflections on dynamic consent in biomedical research: The story so far. European Journal of Human Genetics: EJHG.

[CR49] Thompson R, McNamee MJ (2017). Consent, ethics and genetic biobanks: The case of the Athlome project. BMC Genomics.

[CR50] Usher KJ, Arthur D (1998). Process consent: A model for enhancing informed consent in mental health nursing. Journal of Advanced Nursing.

[CR51] van der Loos KI, Longstaff H, Virani A, Illes J (2015). Consent in escrow. Journal of Law and the Biosciences.

