# Supplementary material for: Towards a Design Toolkit of Informed Consent Models Across Fields: A Systematic Review
Source: Sci Eng Ethics. 2022 Aug 30;28(5):42. doi: 10.1007/s11948-022-00398-x (PMC9427926; doi:10.1007/s11948-022-00398-x)
Supplement: Supplementary file 1 — Additional file 1. Field names. List of meta-fields and sub-fields. [file 11948_2022_398_MOESM1_ESM.docx]

Additional file 1: Field names

| **Fields** | **Sub-fields** |
| --- | --- |
| Biobanking research / genetics and genomics research | Biobanking |
|  | Genetics and genomics research |
| Screening including family and prospective parents | Genetic and epigenetic screening |
|  | Newborn screening |
| Organ donation / transplantation | Organ donation / transplantation (/procurement) |
| Clinical care other / including telehealth and nursing | Telehealth |
|  | Nursing |
|  | Personalized care |
|  | Pediatric care |
|  | Long term care |
| Critical care / critical care research | Critical care |
|  | Critical care research |
| Other clinical research / including pragmatic trials and clinical research on children | Clinical trials |
|  | Pragmatic trials |
|  | Clinical research on children |
| Non-interventional medical research | Health service research |
|  | Epidemiology research / public health research |
|  | Population research |
|  | Secondary research |
|  | Observational medical research |
| Health informatics | Health Information Systems (HIS) |
|  | Electronic Patient Records (EPR) |
|  | Summary Care Reports (SCR) |
|  | Health data |
| ICT and data | Data protection law |
|  | Data |
|  | ICT |
| Other | Criminal law |
|  | Sports research |
